# The Effects and Mechanisms of Flavonoids on Cancer Prevention and Therapy: Focus on Gut Microbiota

**DOI:** 10.7150/ijbs.68170

**Published:** 2022-01-24

**Authors:** Man Wang, Fei Yu, Yuan Zhang, Wenguang Chang, Meng Zhou

**Affiliations:** 1 Institute for Translational Medicine, The Affiliated Hospital of Qingdao University, College of Medicine, Qingdao University, 38 Dengzhou Road, Qingdao, 266021, Shandong, China.; 2 Department of Dermatology, Qilu Hospital (Qingdao), Cheeloo College of Medicine, Shandong University, 758 Hefei Road, Qingdao, 266035, Shandong, China.

**Keywords:** flavonoids, cancer, chemopreventive benefits, gut microbiota, bioactive metabolites

## Abstract

Flavonoids are a group of polyphenolic compounds which are ubiquitously found in plants and are consumed as part of the human diet in substantial amounts. The verification of flavonoids' cancer chemopreventive benefits has led to a significant interest in this field. Gut microbiota includes a diverse community of microorganisms and has a close relationship with cancer development. Increasing evidence has indicated that flavonoids exert anticarcinogenic effects by reshaping gut microbiota. Gut microbiota can convert flavonoids into bioactive metabolites that possess anticancer activity. Here, we present a brief introduction to gut microbiota and provide an overview of the interplay between gut microbiota and cancer pathogenesis. We also highlight the crucial roles of flavonoids in preventing cancer based on their regulation of gut microbiota. This review would encourage research on the flavonoid-intestinal microbiota interactions and clinical trials to validate the chemotherapeutic potentials of targeting gut microbiota by dietary bioactive compounds.

## Introduction

Cancer has become one of the most important causes of human morbidity and mortality around the world 1. Chemotherapy remains the mainstay of cancer treatment 2. However, the use of conventional chemotherapeutic agents has been linked with acquired cancer resistance and various detrimental side-effects. Consequently, significant efforts have been devoted to discovering new compounds and alternative therapeutic options. Natural compounds are attractive candidates for anticancer drug development on the count of their high availability, strong anticancerous efficiency and low toxicity. Among natural compounds, flavonoids have great potential to be developed as anticancer agents, as many studies have documented their important roles in cancer chemoprevention and chemotherapy 3, 4. Flavonoids are a class of polyphenolic compounds in plants responsible for their color, flavor and pharmacological properties 5. Fruits, vegetables, nuts, legumes and plant-derived beverages (e.g., tea and red wine) are main dietary sources of flavonoids. So far, there are over 8000 structurally identified flavonoid molecules 6. Their basic skeletal structure holds 15 carbon atoms that are arranged into a C_6_-C_3_-C_6_ ring system 7. Flavonoids are synthesized in plants as bioactive secondary metabolites in response to stressors and play a critical role in protecting plants from be damaged by pathogens and insects 8. After consumption, flavonoids are mainly metabolized by gut microbiota and host tissues. As a dietary component, flavonoids are found to possess versatile health-promoting properties, including antioxidant, anti-inflammatory, and anticancer effects. According to previous findings, flavonoids exerted anticancer effects via their regulation of cancer-relevant factors and pathways 9. Recently, flavonoids are found to be involved in cancer biology by reshaping gut microbiota 10. Bioactive metabolites produced by gut microbiota from flavonoids exert an inhibitory effect on carcinogenesis 11.

The human gut is colonized by trillions of microorganisms including archaea, bacteria, fungi, protozoa and viruses, which together constitute the gut microbiota 12. The number of gut microbial cells is comparable to that of our own cells 13. Gut microbiota mainly establishes a commensal relationship with the host 13. They constitute an active living population that possesses a metabolic capability similar to the liver. The composition of gut microbiota exhibits wide interpersonal variation but relatively temporal stability in each individual 14. Healthy individuals hold well-balanced, homeostatic gut microbiota. Gut microbiota has been emerged as a crucial component of host metabolism and exerts various physiological functions, such as strengthening the integrity of intestinal mucosal barrier, offering nutrients, protecting against pathogens and orchestrating host immunity 15. Particularly, gut microbiota participates in the pathophysiology of cancer via multiple mechanisms. The oncogenic *Helicobacter pylori* (*H. pylori*) has been found to be closely associated with gastric cancer. Gut microbiota dysbiosis is another critical mechanism associated with carcinogenesis. Gut microbiota dysbiosis contributes to cancer development and progression through induction of DNA damage, production of carcinogenic metabolites, regulation of β-catenin/Wnt signaling and proinflammatory pathways, and suppression of immune system. Therefore, targeting gut microbiota could improve the efficacy of anticancer therapies 16.

In recent years, the impact of flavonoids on gut microbiota has become an active new frontier for cancer research, holding vital keys to understanding the mechanism of anticancer actions of flavonoids. Here, we present an overview of the reciprocal relationship between flavonoids and gut microbiota. Also, we further describe the protective role of flavonoids against cancer via regulating gut microbiota. Although there are multitudinous studies revealing the anticancer mechanisms of action of flavonoids, substantial efforts on gut microbiota are still required to consider flavonoids as promising drug candidates in cancer prevention and treatment.

## Gut microbiota

Gut microbiota refers to the microorganisms inhabiting the human gastrointestinal tract (GIT). This dynamic community is a complex and diverse consortium of microorganisms comprising archaea, bacteria, fungi, protozoa and viruses. Thanks to the availability of multiomics studies and metagenome sequencing, our knowledge of gut microbiota has been rapidly expanding. Approximately 10^14^ microbial cells exist in the human gut, comparable to human cells 17. The human gut microbiota is mostly made up of Actinobacteria, Bacteroidetes, Firmicutes, Fusobacteria, Proteobacteria and Verrucomicrobia, with the two phyla Bacteroidetes and Firmicutes accounting for 90% of the total microbial population 18. Due to the emergence of internal transcribed spacer (ITS) ribosomal sequencing, fungi are also found in gut microbiota. Healthy adult intestinal fungi are dominated by *Aspergillus*, *Candida*,* Debaryomyces*, *Malassezia*, *Penicillium*, *Pichia* and *Saccharomyces* 19. Fungal communities are characterized by low biodiversity and great unevenness compared with bacterial inhabitants. Fungi appear to pass through the GIT without inhabiting the GIT. It is proposed that *Aspergillus* and *Penicillium* are not stable GIT colonizers, but rather environmental or food-borne fungi. Phenotyping of fungal isolates showed that *Candida albicans* clung to human epithelial cells more efficiently and generated greater amounts of biofilm *in vitro* than non-*Candida* fungi 19. Thus, *C. albicans* may be commonly implicated in stable colonization. Additional work is needed to verify if other species identified as potential inhabitants of the GIT, are genuine colonizers or rather reach the intestine spreading from other body districts.

Gut viruses consist of bacteriophages (phages) able to target intestinal bacteria, as well as eukaryotic viruses that replicate in host cells. Phages form the majority of gut viruses. The complexity and diversity of human intestinal phages have been revealed by metagenomics. The families *Myoviridae*, *Podoviridae* and* Siphoviridae* from the order Caudovirales are the major members of intestinal phages 20, 21. Some studies reported the presence of other phage families in the gut, such as *Anelloviridae*, *Circoviridae*, *Inoviridae* and *Microviridae* 22. Phages affect the constitution and diversity of commensal bacteria in the intestine by modulating their mortality, acting as vehicles for horizontal gene transfer, or remodeling host metabolism 23. Intestinal bacteria have evolved various defense mechanisms against phage infection. Generally, bacteria have acquired a sequence-specific adaptive immunity called clustered, regularly interspaced short palindromic repeat (CRISPR) system that protects organisms from invading phages 24. Bacteria are able to hide their membrane receptors to restrict phage docking and replication 25. Bacteria also prevent the dissemination of phages into adjacent cells via the 'abortive infection' mechanism 26. *Anelloviridae* is the most prevalent eukaryotic DNA virus family in the gut, commonly in infants 27. Other eukaryotic viruses, such as *Adenoviridae*, *Geminiviridae*, *Herpesviridae*, *Nanoviridae*, *Papillomaviridae*, *Parvoviridae*, *Polyomaviridae* and *Poxviridae*, have also been detected in the human gut 28. Remarkably, pathogenic viruses (e.g., adenoviruses and herpesviruses) hardly cause diseases and usually stay dormant in the majority of individuals. In addition, gut RNA virome in the human GIT is mainly represented by plant- and insect-related viruses, including* Caliciviridae*, *Picobirnaviridae*, *Picornaviridae* and *Reoviridae* 28.

Enteric viruses and resident bacteria share the same environment within the intestine. As a result, it has been proposed that the interplay between enteric viruses and other components of intestinal microbiota could have an impact on the course of enteric virus infection. Consistently, *in vivo* experiments proved that bacterial inhabitants facilitated the infectivity, propagation and transmission of enteric viruses 29, 30. The promotion effects of microbiota on viral infection involve several mechanisms. The binding of viruses to bacterial components [e.g., lipopolysaccharide (LPS) and histone blood group antigens (HBGAs)] is shown to promote cell attachment and virion stability 31, 32. Moreover, commensal bacteria confer immune evasion to enteric viruses. For instance, bacterial LPS stimulated the production of anti-inflammatory cytokines by inducing the toll-like receptor 4 (TLR4)/interleukin-6 (IL-6) pathway, which favored the enteric persistence of mouse mammary tumor virus (MMTV) in mice 29. Intestinal microbiota inhibited interferon (IFN)-γ-dependent innate immune responses, thus contributing to immune tolerance of mice to norovirus 33. On the contrary, intestinal microbiota can inhibit virus infection by priming antiviral immunity. For example, the commensal bacterium *Blautia coccoides* limited enteric virus replication and pathogenesis by mobilizing type I IFN-mediated antiviral innate immunity 34. Metabolites of gut microbiota also control host immunity and viral infectivity. The previous study showed that *Clostridium orbiscindens*-produced metabolite desaminotyrosine could protect mice from influenza virus infection by activating type I IFN-mediated immune responses 35. Indigenous commensal bacteria even block virus infection independent of host immune system in some circumstances. It turned out that *Bacillus subtilis*-produced peptide P18 exerted an inhibitory effect on influenza virus invasion in a murine infection model 36. Thus, commensal microbiota plays dual roles in controlling enteric virus infection.

Enteric viruses have an impact on the host immune system. Gut viruses, especially phages, are able to trigger host innate immunity and thus protect against pathogenic infections 37. The host immune system in turn has a key role in dominating the composition and expansion of resident viral communities in the intestine 38. Furthermore, commensal viruses can control intestinal inflammation and contribute to intestinal homeostasis through TLR-mediated anti-inflammatory cytokine production 39. Currently, the study on intestinal viruses is in its initial stage and there are still many gaps in our knowledge with regard to this field. Particularly, standardized approaches for gut virome analysis are still lacking, and it is thus challenging to differentiate intrahost viral populations in the intestine from those responsible for acute infection. Considerable efforts must be made to characterize the gut virome and to decipher the reciprocal crosstalk among intestinal microbes. In addition, longitudinal researches aimed at exploring the dynamics of the gut virome will be critical to advance our understanding of the exact mechanisms involved in the impact of gut microbiome on human health and disease.

Human gut microbiota develops from birth to early childhood and becomes stable in adults 40. Multiple factors, including host genotype, health status, delivery mode, dietary habit, age and gender, may give rise to dramatic variability in the composition of gut microbiota from individual to individual 41. Additionally, it should be noted that the composition of human gut microbiota constantly alters in some circumstances, such as dietary variation, antibiotic exposure, exercise, drug usage, sleep loss and stress 42. Intriguingly, the initial microbiota can usually reshape itself upon restoring the original condition. Gut microbiota has been shown to orchestrate several physiological functions, including metabolism of nutrients, maintenance of the intestinal barrier, protection from pathogens, inflammation and immunity 42.

## Gut microbiota and cancer

**Figure 1:**
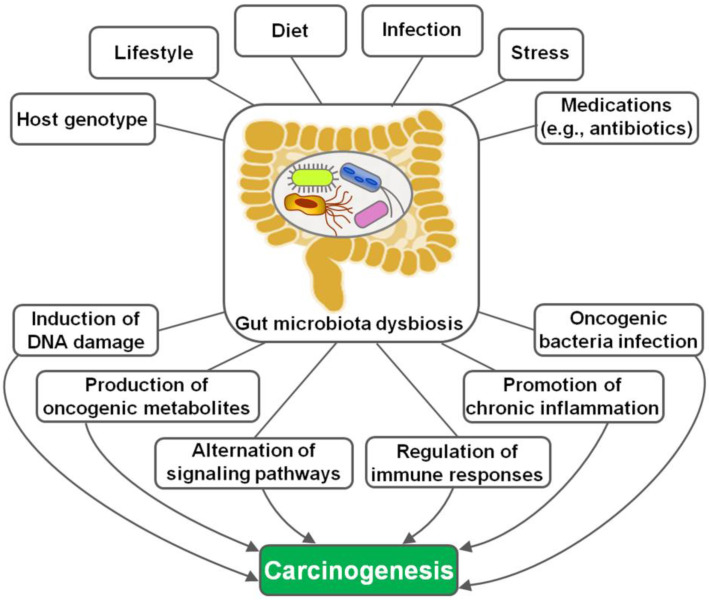
** The mechanisms underlying gut microbiota dysbiosis-induced carcinogenesis.** Host genotype, lifestyle, diet, infection, psychological stress and medications are important factors leading to intestinal dysbiosis. Gut microbiota dysbiosis drives carcinogenesis via different mechanisms, including induction of DNA damage, production of tumor-promoting metabolites, regulation of diverse signaling pathways, manipulation of host immune responses, activation of chronic inflammation and promotion of oncogenic bacteria infection.

The implication of gut microbiota in disease pathogenesis has been gradually understood. In recent years, the interaction between gut microbiota and cancer has attracted a great deal of attention in the efforts to characterize the detailed mechanisms through which gut microbiota is involved in cancer development. Remarkably, microbial dysbiosis is believed to be a predominant factor contributing to carcinogenesis (Figure 1) 16. Intestinal dysbiosis involves the qualitative and quantitative variations of gut microbiota that result in disruption of physiological homeostasis of intestinal epithelial cells 43. Diet changes, antibiotic therapies and inflammatory intestinal diseases are common causes of intestinal dysbiosis. Dysbiosis may lead to a breach in the mucosal barriers. Once mucosal barriers are permanently breached, microbiota may affect carcinogenesis through diverse mechanisms, including induction of DNA damage, production of carcinogenic metabolites, alternation of signaling pathways and regulation of host immune responses. Besides, oncogenic bacteria within gut microbiota are one of the contributory factors for carcinogenesis.

## Gut microbiota and colorectal cancer

Colorectal cancer (CRC), also known as colon cancer or bowel cancer, represents the third most common malignancy worldwide 44. The occurrence and progression of CRC are intricate processes that involve multitudinous environmental, genetic and epigenetic parameters. The gut microbiota has been regarded as an important environmental factor contributing to CRC pathogenesis. Structural alternation of gut microbiota is closely tied to the development of CRC. So far, several mechanisms underlying the impact of gut microbiota on CRC development have been identified, which will be discussed in detail below.

### DNA damage

Bacteria-derived toxins can directly cause DNA damage. The accumulation of DNA damage leads to the occurrence of oncogenic mutations. Colibactin, produced by members of the *Enterobacteriaceae* family, promoted the overgrowth of intestinal epithelial cells and contributed to colorectal carcinogenesis by inducing DNA damage, mutation and genomic instability 45. Cougnoux et al. 46 revealed that small molecular inhibitors of ClbP, an enzyme involved in colibactin synthesis, were capable of preventing the genotoxic activity of colibactin-producing *Escherichia coli* and thus suppressed colorectal tumorigenesis.

Cytolethal distending toxin (CDT) produced by *Campylobacter jejuni* triggered genomic instability and the acquisition of malignant traits in colon epithelial cells 47. Sulfidogenic bacteria-produced hydrogen sulfide was a potent genotoxin that induced DNA damage and might have a close relationship with CRC pathogenesis 48. Deoxycholic acid (DCA) functions as a potential pro-carcinogenic agent by inducing DNA damage, aneuploidy, micronuclei formation and genomic instability 49. CRC patients had higher fecal or serum levels of DCA than healthy subjects, suggesting the linkage between DCA and the etiology of CRC 50. Bacterial toxins also indirectly damage host DNA. For instance, enterotoxigenic *Bacteroides fragilis* toxin (BFT) exposure stimulated spermine oxidase (SMO)-dependent generation of reactive oxygen species (ROS) and led to DNA damage in intestinal epithelial cells 51. Thus, BFT plays an important role in *B. fragilis*-induced colon tumorigenesis.

### Metabolism

Accumulating evidence has proven that human gut microbiota participates in multiple processes associated with cancer etiology via its metabolites. For instance, lactate, an intermediate and/or terminal metabolite produced by gut microbiota, stimulated angiogenic responses for delivering oxygen, glucose and other nutrients to colon cancer cells, thus promoting CRC cell proliferation, migration and invasion 52. Protein-derived metabolites phenylacetic acid, phenol, 4-hydroxyphenylacetic acid and acetaldehyde showed cytotoxic or genotoxic effects on colon cells 53. Xu et al. 54 revealed a potential association between trimethylamine N-oxide (TMAO), an intestinal microbial metabolite of fat and red meat, and the risk of CRC. High-protein diet causes the increased production of N-nitroso compounds (NOCs) by gut microbiota. A large population-based case-control study demonstrated that NOC intake was associated with a higher risk of CRC 55.

It is known that colonic mucosa is continually exposed to gut microbiota and its metabolites. Gut microbiota is implicated in carcinogenesis through metabolites and molecules that affect host immune cell responses 56. Short-chain fatty acids (SCFAs) released from gut microbiota inhibited inflammatory responses through activation of the G protein-coupled receptor (GPCR) signaling or inhibition of histone deacetylases (HDACs) 57. Peptidoglycan generated by gut microbiota was shown to stimulate the innate immune system through nucleotide-binding oligomerization domain 1 (NOD1) 58. Polyamines in mammals are mainly derived from diet, endogenous biosynthesis and gut microbiota metabolism of amino acids. Polyamines could perturb antitumor immune function in CRC by reducing the expression of adhesion molecules (e.g., CD44 and LFA-1) and cytokines (IFN-γ and TNF) 59.

### Regulation of diverse signaling pathways

Gut microbiota is able to affect signal transduction pathways that have been widely implicated as a regulator of cell proliferation, migration and invasion. For instance, DCA-mediated intestinal dysbiosis damaged the intestinal barrier function, affected the levels of cytokines and chemokines in the tumor microenvironment and eventually facilitated intestinal carcinogenesis via activation of the Wnt/β-catenin signaling pathway 60. Gut microbiota-derived LPS expedited CRC cell growth by activating the c-Jun/c-Jun N-terminal kinase (JNK) signaling pathway 61. Gut microbiota functions to stimulate proinflammatory pathways involved in the induction of carcinogenesis. Specifically, *Fusobacterium nucleatum* infection enhanced the proliferative and invasive capabilities of CRC cells by activating the nuclear factor-κB (NF-κB) signaling pathway 62. Conversely, silencing of NF-κB impaired the oncogenic effects of *F. nucleatum* on CRC cells. Putative cell wall-binding repeat 2 (PCWBR2), a surface protein of *Peptostreptococcus anaerobius*, directly combined with integrin α2/β1, which was usually overexpressed in human CRC cells 63. This interaction promoted CRC development by inducing NF-κB-mediated proinflammatory response. Enterotoxigenic* B. fragilis* triggered colitis-associated colon carcinogenesis by stimulating signal transducer and activator of transcription 3 (STAT3)-mediated T helper 17 (Th17) responses 64.

## Gut microbiota and gastric cancer

### Helicobacter pylori

*H. pylori* is one of the most ubiquitous bacterial pathogens, which occupies the stomach mucosa and alters gastric microbiota leading to a spectrum of gastric diseases. *H. pylori* has been proven to be implicated in gastric cancer and is categorized as a group I carcinogen by World Health Organization (WHO) 65. *H. pylori* produces a diversity of virulence factors that interfere with intracellular signaling mechanisms, hence facilitating cancer development. Among these virulence factors, oncoproteins cytotoxin-associated gene A (CagA) and vacuolating cytotoxin A (VacA) are identified as crucial virulence factors that are intertwined with the pathogenesis of gastric cancer, which will be discussed in detail herein.

It was reported that infection with CagA-positive *H. pylori* strains significantly increased the risk of gastric cancer 66. The mechanisms involved in CagA-induced gastric carcinogenesis were previously elucidated. Several studies revealed CagA-positive *H. pylori*-induced activation of the epithelial mesenchymal transition (EMT) process within the gastric mucosa, which was associated with cell motility, invasiveness and metastasis during carcinogenesis 67, 68. Further investigations showed that CagA-positive *H. pylori* infection caused the aberrant expression of key molecules associated with EMT (e.g., E-cadherin, N-cadherin, vimentin, Slug and Snail), the attenuation of glycogen synthase kinase-3 (GSK-3), activation of fibroblasts, induction of cancer stem cell (CSC)-like properties or regulation of the yes-associated protein (YAP) pathway 69, 70.

CagA represses the activity of tumor suppressors. The expression of gastrokine 1 (GKN1) was decreased in *H. pylori*-infected gastric mucosa and gastric cancer 71. The downregulation of GKN1 was mediated by the activation of CagA-induced extracellular signal-regulated kinase (ERK)/AU-rich element RNA-binding factor 1 (AUF1) pathway during *H. pylori* infection. Thus, CagA facilitated the progression of gastric cancer partially by reducing GKN1 expression. A recent report indicated that CagA could target another tumor suppressor, apoptosis-stimulating protein 2 of p53 (ASPP2) 72. The association between CagA and ASPP2 damaged the cellular polarity of infected gastric epithelial cells and endowed cells with EMT-like properties. Reportedly, individuals infected CagA-positive *H. pylori* presented a higher probability of compassing p53 mutations, which could perturb p53 tumor suppressor pathways 73. Conversely, tumor-promoting pathways, such as ERK/mitogen-activated protein kinase (MAPK), NF-κB, phosphatidylinositol 3-kinase (PI3K)/protein kinase B (Akt), Ras, STAT3 and Wnt/β-catenin, was activated in CagA-positive *H. pylori*-infected cells. The profound impact of CagA on the pathways mentioned above can cause many consequences, such as induction of cell proliferation, malignant transformation and cell invasion.

Gastric cancer is deemed as an inflammation-related cancer. *H. pylori* infection-associated chronic inflammation in the gastric mucosa is a principal step in the occurrence and development of gastric cancer. CagA-positive *H. pylori* increased the levels of many inflammatory cytokines in the stomach of infected individuals, encompassing IFN-γ, tumor necrosis factor-α (TNF-α), IL-1, IL-6, IL-8, IL-10 and IL-18. This resulted in the activation of multiple types of immune cells, such as dendritic cells, mast cells, lymphocytes, macrophages and neutrophils. Furthermore, CagA orchestrated several signal transduction pathways [e.g., NF-κB and c-Met-PI3K/Akt-mammalian target of rapamycin (mTOR) signalings] that led to inflammatory responses 74, 75. CagA-stimulated inflammatory response constitutes important mechanisms underpinning gastric inflammation and carcinogenesis.

VacA plays an important role in bacterial colonization and survival in the gastric epithelium. VacA induced cell vacuolation, and participated in autophagy, apoptosis and necrosis within gastric epithelial cells 76. Similar to CagA, VacA was capable of controlling a diversity of signaling cascades, such as MAPK, EKR, Wnt/β-catenin and PI3K/Akt pathways 77. In addition, VacA markedly influences the function of immune cells, which involved its contribution to suppression of B/T cell activation and expansion, induction of macrophage apoptosis via blockade of the IFN-β signaling, elevated secretion of IL-8, as well as stimulation of regulatory T cell differentiation 78. These features are connected with immune dysregulation and gastric mucosal damage, both of which can contribute to gastric carcinogenesis.

Apart from the above-discussed mechanisms, other factors also have an impact on the course and development of *H. pylori*-induced gastric carcinogenesis. For instance, *H. pylori*-derived LPS activated the TLR4 signaling pathway in mononuclear cells and further repressed T cell-mediated cytotoxicity, contributing to the onset and progression of gastric cancer 79. Oxidative stress in the gastric mucosa due to *H. pylori* infection was associated with significant damage of the gastric mucosa, hence resulting in the pathogenesis of gastric cancer 80. Furthermore, host genetics and some environmental factors (e.g., diet) act as pivotal contributing factors to gastric carcinogenesis 81. Collectively, *H. pylori*-induced gastric carcinogenicity is a consequence of intricate interplays among bacterial virulence factors, host and environmental factors. The underlying mechanisms of *H. pylori*-caused gastric cancer have not been fully defined, and further work is warranted.

### Non-Helicobacter pylori microbiota

Some bacteria other than *H. pylori* in the stomach also play a role in the development of gastric cancer. Dysbiosis of gastric microbiota has a potential relationship with the occurrence of gastric cancer. The previous study indicated that patients with gastric cancer had different gut microbiota community structures 82. The abundance of *Achromobacter*, *Citrobacter*, *Clostridium*, *Lactobacillus*, *Phyllobacterium* and *Rhodococcus* was increased in patients with gastric cancer compared with those with chronic gastritis. It appeared that these commensal microbes could be opportunistic pathogens. A cohort study involving 268 patients with gastric cancer and 288 healthy controls showed that individuals who carried higher abundances of *Prevotella copri* and *Propionibacterium acnes* exhibited a markedly higher risk of gastric cancer than non-carriers 83. Moreover, it was found that *P. copri*- or *P. acnes*-induced inflammatory responses might be associated with gastric carcinogenesis 84, 85. Altogether, bacteria exhibiting an elevated abundance may be involved in the etiology of gastric cancer, but the exact mechanisms are worthy of further study.

## Gut microbiota and liver cancer

Gut microbiota have been linked with hepatocellular carcinoma (HCC). Through the portal venous system, the liver is commonly exposed to intestinal bacterial components and their metabolites, which may induce inflammatory changes and hepatotoxicity, eventually contributing to liver carcinogenesis. For instance, altered gut microbiota increased the concentration of hepatic bile acids and thus drove liver carcinogenesis in a mouse model of obesity-induced HCC 86. Dietary or genetic obesity changed the composition of gut microbiota, thus increasing the production of pro-tumorigenic DCA 87. DCA induced senescence-associated secretory phenotype in hepatic stellate cells, which secreted multiple inflammatory and pro-tumorigenic molecules in the liver. These events promoted HCC progression in mice following exposure to chemical carcinogen. On the contrary, blockade of DCA generation suppressed HCC development in obese mice. These findings shed light on underlying mechanisms associated with obesity-associated carcinogenesis.

## Gut microbiota and breast cancer

The linkage between gut microbiota and breast carcinogenesis was previously investigated. The high abundance of Bacteroidetes, *Blautia* spp., *Clostridium coccoides*, *Clostridium leptum* and *Faecalibacterium prausnitzii* was correlated with advanced clinical stage in patients with breast cancer 88. A population-based case-control pilot study showed that patients with breast cancer had higher abundances of *Clostridiaceae*, *Faecalibacterium* and *Ruminococcaceae*, and lower levels of *Dorea* and *Lachnospiraceae* than control subjects 89. The altered composition and diversity of gut microbiota have a potential impact on the development of breast cancer. Gut microbiota was proposed to promote breast carcinogenesis by affecting the level of circulating estrogens, energy metabolism, obesity, or antitumor immune function 90. However, only limited supporting evidence is available. Considerable efforts are needed to clarify the roles of gut microbiota in breast cancer formation.

## Gut microbiota and urogenital system tumor

Dysbiosis of gut microbiota may be associated with the carcinogenesis of urogenital system tumor. Compared with healthy controls, the levels of *Clostridium* cluster XI and *Prevotella* were reduced in bladder cancer patients 91. Several studies compared the composition of gut microbiota between patients with cervical cancer and healthy controls. It turned out that cervical cancer patients had a high abundance of Bacteroidetes and a low level of Firmicutes compared with controls 92. Another study indicated that *Dialister*, *Porphyromonas* and *Prevotella* were enriched, whereas *Alistipes*,* Bacteroides* and* Lachnospiracea* showed a relatively low abundance in cancer group compared with the control group 93. In a recent study, a higher level of *Prevotella* was found in patients with cervical cancer than in healthy controls 94. It was presumed that gut microbiota influenced cervical cancer development possibly through activation of inflammatory responses, but this assumption remained to be validated. Further mechanistic investigations are required to verify the specific association between gut microbiota and cervical oncogenesis.

The previous study revealed an increased in the count of proinflammatory *Bacteroides* and *Streptococcus* in patients with prostate cancer compared with healthy controls 95. The numbers of *Alistipes*, *Lachnospira*, *Rikenellaceae* and SCFA-producing bacteria were positively associated with a high risk of prostate cancer 96. It is likely that dysbiosis of gut microbiota may cause inflammation and neoplastic events even at a systemic level. Gut microbiota could contribute to prostate cancer development by influencing the metabolism of specific compounds that may be linked to increased prostate cancer risk. For instance, *Clostridium* could transform glucocorticoids into androgens in the gut, which facilitated the development of prostate cancer 97. Gut microbiota-derived SCFA upregulated local and systemic insulin-like growth factor-1 (IGF-1), which favored prostate cancer development 98. *Ruminococcus*, which was involved in glycerolphospholipid metabolism, promoted prostate cancer progression by upregulating lysophosphatidylcholine acyltransferase 1 (LPCAT1) and activating DNA repair pathways 99. At present, the detailed mechanisms through which gut microbiota affects prostate cancer progression are elusive. A great deal of further work is necessary to fully understand the relationship between gut microbiota and prostate cancer pathogenesis.

The complex and bidirectional relationship between gut microbiota and carcinogenesis has received considerable attention in recent years. Intestinal microbes and cancer cells co-evolve in the body's ecosystem, and they may compete with each other for incoming resources necessary to survival and replication 100. Daily diet, energy or nutrients may influence the growth of both microbial cells and cancer cells. Microbes and cancer cells can interact with each other in multifaceted ways that affect their survival and proliferation 101. Many studies have demonstrated altered gut microbiota profiles in cancer patients. *Bifidobacterium*, *Blautia* and *Faecalibacterium* were found in lower proportion in gut microbiota of CRC patients than healthy subjects, whereas *Fusobacterium*, *Mogibacterium* spp., *Peptostreptococcus* and *Porphyromonas* were abundant in fecal samples from CRC patients 102, 103. Coker et al. noted an enrichment of the phylum Fusobacteria and the genera *Dialister*, *Mogibacterium* and *Peptostreptococcus* in gastric cancer compared with other disease stages, including superficial gastritis, atrophic gastritis and intestinal metaplasia 104. Patients with gastric adenocarcinoma had higher abundance of *Enterobacteriaceae* and lower abundance of *Barnesiella*, *Bifidobacterium*, *Lachnoclostridium* and *Parabacteroides* in comparison with healthy controls 105. Previous studies revealed significantly higher abundance of intestinal microbes belonging to the Phyla Actinobacteria, Bacteroidetes, Firmicutes, Fusobacteria, Proteobacteria and Verrucomicrobia, as well as the genera *Bifidobacterium*, *Porphyromonas* and *Prevotella* in patients with pancreatic cancer than in healthy controls 106-108. Despite the verification of the changes in intestinal microbiota composition in cancer patients, these studies do not provide sufficient evidence of whether the dysbiosis of gut microbiota acts causatively or consequently in cancer pathogenesis.

On the other hand, there is evidence indicating that gut microbial populations play dual roles in cancer pathogenesis. Commensal microbes may exert anticancer effects through protection against gut dysbiosis, bioconversion of chemotherapeutic agents or enhancement of anticancer immunity 109. Conversely, some resident bacteria (e.g., *B. fragilis* and *F. nucleatum*) rise during gut dysbiosis and induce cancer genesis 110. Meanwhile, various experimental studies have suggested the causal linkage between intestinal dysbiosis and cancer development. For instance, preclinical studies showed that transplant of feces from CRC patients stimulated polyp formation, triggered procarcinogenic signals and affected the local immune environment in recipient mice 111. Reportedly, there were striking and continuous alterations in the composition of gut microbiota during cancer development in a murine model of azoxymethane (AOM)/dextran sulfate sodium (DSS)-induced CRC 112. Furthermore, the depletion of gut microbiota by an antibiotic cocktail could significantly inhibit CRC growth in mice. Dysbiosis of gut microbiota caused by environmental changes (e.g., diet, infection, or lifestyle) may contribute to the occurrence and development of cancer by triggering inflammation, promoting cell proliferation, facilitating tumor immune evasion or altering anticancer drug metabolism 109. Collectively, cancer development can modify the structure of gut microbiota, but, vice versa, changes of intestinal microbes also influence cancer pathogenesis. It should be noted that although the connection between gut microbiota and carcinogenesis has been established, these associations have yet to be well defined and warrant further investigation. It is still unclear whether carcinogenesis is the cause or consequence of the changes in gut microbiota. Future ongoing studies are needed to fully clarify the complicated relationship between gut microbiota and cancer.

## Flavonoids

### Food sources and classification of flavonoids

**Figure 2:**
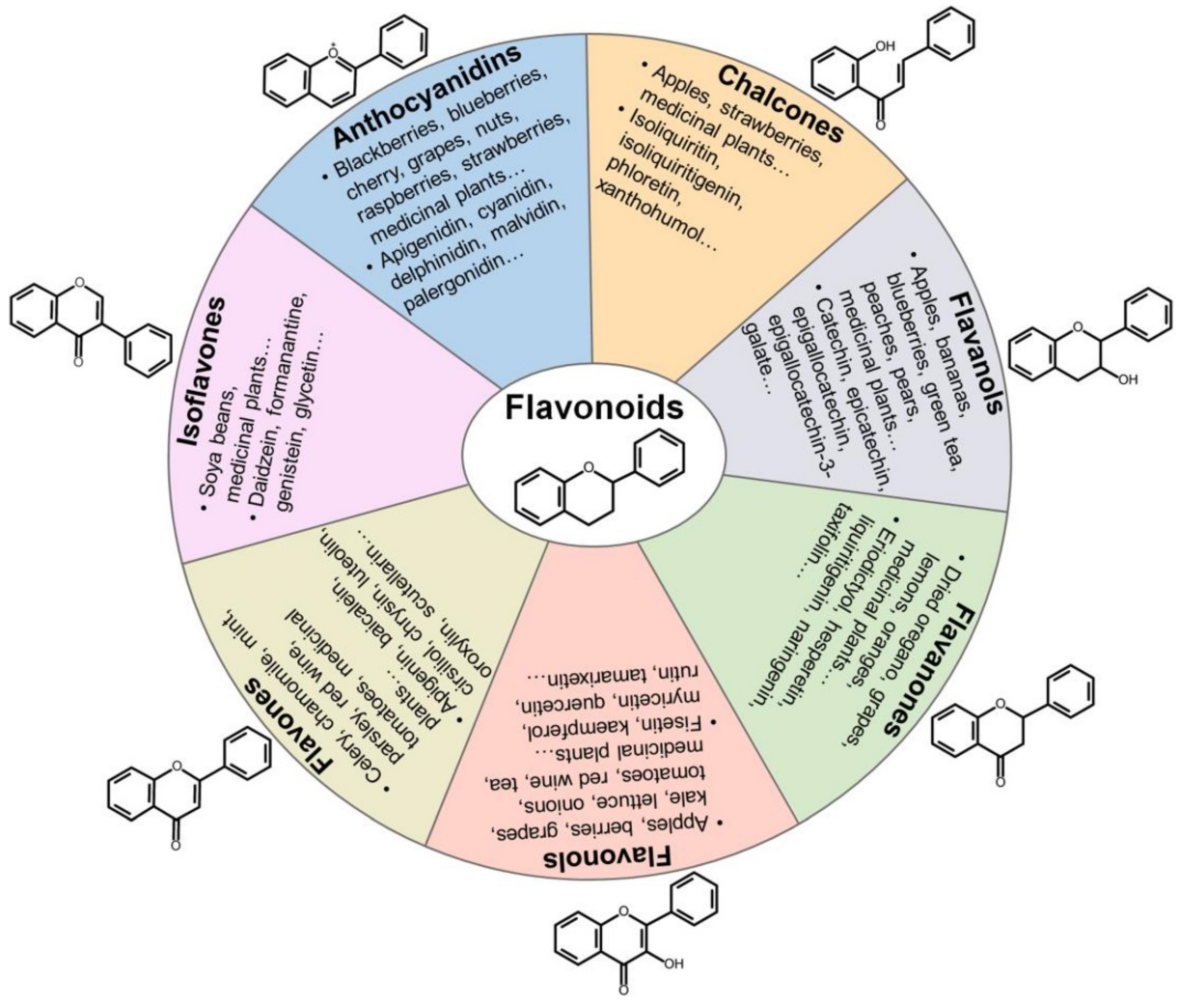
**Categorization, food sources, representatives and chemical structures of flavonoids.** Flavonoids are naturally present in fruits, vegetables and plant-derived beverages. Based on their chemical structures, flavonoids are generally classified into seven main groups including anthocyanidins, chalcones, flavanols, flavanones, flavonols, flavones and isoflavones.

Flavonoids are polyphenolic compounds synthesized in fruits and vegetables as bioactive secondary metabolites responsible for their color, flavor and pharmacological properties 8. Flavonoids function to protect plants against bacteria and radiations. Flavonoids are composed of two aromatic carbon rings joined by a three-carbon chain that forms an oxygenated heterocyclic ring (Figure 2). Among the fruits, the highest levels of flavonoids are found in apples, berries, cherries and plums 113. Among the vegetables, broad beans, olives, onions, shallot and spinach are the richest in flavonoids. Tea, wine and some medicinal herbs are also the rich source of flavonoids. Flavonoids exert many biological functions, including antioxidant, antimicrobial, anti-inflammatory and anticancer activities.

Flavonoids can be grouped into distinct classes based on their molecular structures: anthocyanidins, chalcones, flavanols, flavanones, flavonols, flavones, and isoflavones (Figure 2). Anthocyanidins are water-soluble natural pigments that are widely found in fruits and plant petals. Cyanidin, delphinidin, malvidin, pelargonidin, peonidin and petunidin are the most prevalent anthocyanidins in plants 114. Chalcones are one of the main classes of flavonoids whose colors can change from yellow to orange and serve as open-chain precursors for synthesis of flavonoids and isoflavonoids 115. Chalcones, mainly represented by isoliquiritin and isoliquiritigenin (ISL), are abundant in edible plants including apples, citrus, nuts, potatoes, shallots, tomatoes. Apples, berries, cocoa, grapes, green tea and red wine are among the main sources of flavanols. Flavanols exist in both the monomer form (catechins) and the polymer form (proanthocyanidins). Catechin and epicatechin are main flavanols in fruits, while epigallocatechin, epigallocatechin gallate (EGCG) and gallocatechin are common flavanols present in tea and grapes 116. Flavanones are polyphenols specific of citrus fruits, where they are present at high concentrations 114. Flavanones are principally represented by eriodictyol, hesperetin, naringenin and neohesperidin. Moreover, flavanones have been found to possess various pharmacological activities, including antimicrobial, antitumor, antioxidant and anti-inflammatory properties. Flavonols are the most prevalent flavonoids in many plant species. They have been found in apples, berries, broccoli, onions, grapes, red wine and teas 114. Flavonols are biosynthesized from dihydroflavonols through the action of flavonol synthase. The most common types of flavonols are isorhamnetin, kaempferol, myricetin, quercetin and rutin. Flavonols possess prominent antioxidant and antimicrobial functions 117. Flavones are primarily found in celery, chamomile tea and garlic 113. Flavones are represented by apigenin, baicalein, luteolin and polymethoxyflavones (PMFs). Isoflavones are mainly found in leguminous plants and are present as aglycones or glycosides 118. Soy and its products are the principal source of isoflavones in the human diet. Daidzein and genistein are two prominent isoflavones in soy. Formanantine and glycetin are also common isoflavones.

## Metabolism and bioavailability of flavonoids

Flavonoids are generally absorbed via dietary intake. Average flavonoid intake varies from 60 to 600 mg per day 119. Flavonoids are mainly present in their glycosidic forms that have low lipophilicity and cannot be directly absorbed in the small intestine. This feature results in the poor absorption and low bioavailability of flavonoids. Flavonoids can only be absorbed after removal of conjugated glycosyl. The type, number and position of linked sugars may be important factors influencing the absorption of flavonoid glycosides in the small intestine. The breakdown of flavonoids is commonly mediated by gut microbiota 120. After consumption, intestinal microbiota catalyzes the hydrolysis of glycosylated flavonoids including anthocyanins, flavones, flavonols and isoflavones into their respective aglycones, which are then transported to intestinal epithelial cells through passive diffusion. Glycosidases produced by intestinal bacteria perform the hydrolysis. Following absorption, flavonoids undergo metabolic transformation in the small intestine, liver and kidney. In intestinal epithelial cells, flavonoid aglycones undergo phase I and II metabolism, producing various glucuronidated, sulfated and methylated conjugates 120. These resultant metabolites produced by intestinal microbiota are transported to the liver via the portal vein, where they are subjected to further conjugation reactions. Conjugation can benefit the excretion of flavonoids and thus shortens their plasma half-life. The efflux of flavonoids from the human body is mainly via renal, biliary and fecal excretion 121. Particularly, flavonoid metabolites (e.g., glucuronides and sulfates) can be excreted back into the duodenum via the biliary route, where they are hydrolyzed to aglycones by microbial enzymes (glucuronidases and sulfatases) 122. Aglycones produced by the hydrolysis reactions may be reabsorbed and undergo additional rounds of enterohepatic recycling.

A large fraction of dietary flavonoids remains unabsorbed along the gastrointestinal tract and reach the large intestine where they are subjected to the action of intestinal microbiota. Colonic metabolism plays a major role in the overall metabolism of flavonoids and the conjugated metabolites that are excreted back into the intestine lumen via enterohepatic circulation 120. Intestinal bacteria-produced enzymes can perform a variety of reactions, such as deglycosylation, dihydroxylation, demethylation and oxidation. The colonic biotransformation of flavonoids results in the production of aromatic catabolites and small phenolic acids. The health-promoting effects of flavonoids are likely to be attributed more to phenolic metabolites formed by colonic metabolism, rather than to their original form. It has been reported that flavonoids have the ability to remodel gut microbiota, which in turn has an influence on the absorption of flavonoids in the intestine 123. However, the bidirectional linkage between flavonoids and gut microbiota has not yet been well understood. Further study is necessary to adequately elucidate these reciprocal interactions.

The highest concentration of flavonoid metabolites in human plasma is generally reached 1 to 2 h after the consumption of flavonoid-rich foods. Flavonoids have poor intestinal bioavailability and rapid excretion, and thus the plasma concentrations of flavonoids rarely exceed 1 μM in individuals on a regular diet 124. There are differences in bioavailability among distinct kinds of flavonoids. According to previous studies, isoflavones have the best rate of absorption, followed by flavanols, flavanones, flavonols, proanthocyanidins and anthocyanins 125. The absorption rate of anthocyanins was reported to be lower than 1% in subjects after consumption of commercial black currant juice 126. Nevertheless, the bioavailability of anthocyanins might be underestimated due to hurdles in detecting anthocyanin metabolites. Anthocyanins and flavanols show the most rapid excretion rates. Plasma anthocyanins reached the maximal level within 1-4 h after intake, and anthocyanins achieved the highest levels in urine at approximately 2.5 h 127. Plasma half-life for anthocyanins ranged from 2 to 3.3 h. Catechin metabolites were excreted very rapidly, having a half-life of only 2-3 h in plasma 128. The majority of catechin metabolites were eliminated during the first 4 h after ingestion. The half-life for hesperetin was estimated to be 3 h and for naringenin about 2.5 h, respectively 129. The peak plasma level of quercetin metabolites in human was reached 2.9 h after onion consumption. The half-life of their efflux phase was 16.9 h 130. Compared to other flavonoids, flavonols had relatively slow excretion rates.

## The impact of flavonoids on gut microbiota

Flavonoids can alter the composition of gut microbiota by increasing the abundance of beneficial organisms and reducing the abundance of harmful species. Therefore, flavonoids improve the gut health by inhibiting the production of endotoxin, driving the conversion of primary into secondary bile acids, preserving gut immune homeostasis, and facilitating nutrient absorption 131. Anthocyanins are capable of causing compositional variations in gut microbiota. *In vitro* gastrointestinal digestion and fecal fermentation revealed that blueberry anthocyanin extract markedly increased the abundance of Bacteroidetes and decreased that of Firmicutes 132. A low abundance of *Enterobacteriaceae* and a high abundance of *Bacteroidaceae*, *Phascolarctobacterium* and *Sutterella* were observed in the fecal microbiota fermented with anthocyanins/flavonol glycosides 133. The amount of *Lactobacillaceae* was decreased, while the count of *Clostridiaceae* was increased in both ileal and colonic lumen of piglets fed with grape seed anthocyanidins 134. Another *in vivo* study demonstrated that bilberry anthocyanins induced the growth of beneficial bacteria (e.g., *Aspergillus oryzae*, *Bacteroides*, *Clostridiaceae-1* and *Lactobacillus*) and inhibited the growth of harmful bacteria (e.g., Euryarchaeota and Verrucomicrobia) 135.

Epicatechin and catechin increased the levels of probiotics (e.g., *Bifidobacterium* spp., *Eubacterium rectale*-*C. coccoides* and *Lactobacillus*) 136. Likewise, the *in vivo* experiment indicated that intake of cocoa flavanols increased the population of *Bifidobacterium* spp. and *Lactobacillus* 137. Analysis of microbial community compositions in the *in vitro* fecal fermentation of theaflavin-3,3'-digallate (TFDG) and EGCG showed a rise in the levels of *Bacteroides* and *Lachnoclostridium* and a decline in the abundance of *Prevotella* 138. Similar results were observed in several studies, which revealed that intake of hesperidin and naringin could improve gut microbiota homeostasis by elevating the abundance of *Bifidobacterium* spp. and *Lactobacillus* spp. 139, 140. Hesperidin supplementation increased the amount of *Bacteroides*/*Prevotella*,* E. coli*, *Staphylococcus* and *Streptococcus*. Similarly, PMFs administration led to a significant increase in the numbers of *Bifidobacterium* and *Lactobacillus* in mice 141. Quercetin suppressed the *in vitro* growth of several pathogens, including *Bacillus cereus*, *H. pylori*, *Listeria monocytogenes* and *Salmonella enteritidis* 142.

The aforementioned studies provide evidence for the impact of flavonoids on the composition and diversity of gut microbiota. It is worth noting that most studies only suggested the general effects of flavonoids on specific bacterial phyla and genera. Currently, there is a significant gap in our knowledge about how flavonoids improve intestinal microbiota. It was speculated that quercetin might change bacterial abundance by affecting the expression of the genes or enzymes involved in metabolic processes 143. Flavonoids are adequately metabolized in the gut, hence producing a variety of metabolites. Further research is essential to identify the molecular targets of flavonoid metabolites and their exact roles in regulation of the composition and abundance of gut microbiota. Compounds from the same category of flavonoids may have diverse effects on the same bacterial strain. Inter-individual variability in gut microbiota may cause the generation of different flavonoid metabolites, leading to distinct responses to the same flavonoid among individuals. The interaction between flavonoids and other dietary components could interfere with the colonic metabolism of flavonoids. For instance, fermentable fibers were shown to alter the manner in which rutin was metabolized by the action of colonic microbiota 144. Thus, it is far too early to draw conclusions concerning the molecular mechanisms of action of flavonoids on gut microbiota based on existing evidence.

## Targeting gut microbiota with flavonoids on cancer prevention and treatment

**Figure 3:**
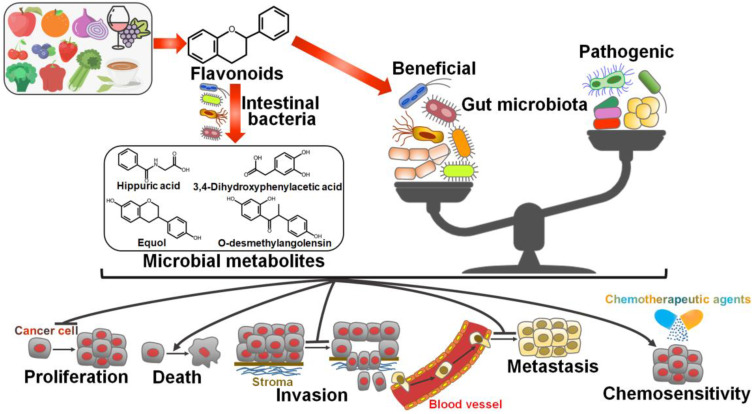
** Schematic illustration of the beneficial effects of flavonoids against cancer via regulating gut microbiota.** Flavonoids ameliorate gut microbiota dysbiosis by elevating the abundance of beneficial microbial organisms and reducing the counts of opportunistic pathogenic species. As a result, flavonoid-mediated modulation of gut microbiota contributes to prevention of cancer cell proliferation, invasion and metastasis. Flavonoids also promote cancer cell death and enhance chemotherapeutic sensitivity of cancer cells by reverting imbalanced gut microbiota. Furthermore, gut microbiota can transform flavonoids into bioactive metabolites that show anticancer activities.

It is well documented that gut microbiota has a close relationship with cancer biology. Therefore, modulation of gut microbiota composition has been deemed as a critical mechanism for flavonoid-mediated cancer chemoprevention (Figure 3).

## Flavonoids, gut microbiota and colorectal cancer

It was proven that anthocyanidin treatment significantly suppressed CRC development in mice (Table 1) 145. Probiotic bacteria can convert carcinogens into less toxic metabolites, and phase I/II enzymes are involved in this process. Enterotoxigenic *B. fragilis* was found to cause dysfunction of the balance between phase I and II enzymes in colon tissue 145. Importantly, anthocyanidins mitigated *B. fragilis*- or carcinogen-induced shift in phase I/II enzyme expression, hence inhibiting the growth of colon tumor in *Apc*^Min/+^ mice. The regulation of phase I and II enzymes was attributed to anthocyanidin-induced deregulation of Aryl hydrocarbon receptor (AhR) and AhR repressor (AhRR). Collectively, gut microbiota dysbiosis and exposure to carcinogens could disturb the balance between phase I and II enzymes in colon tissue. Anthocyanidins might contribute to a state of detoxification in *B. fragilis*-infected cells or environmental carcinogen-exposed cells by reversing the imbalance in expression levels of both phase I and phase II enzymes. The anticancer property of anthocyanidins may be mediated through their effects on carcinogen metabolism. This study reinforced the roles of anthocyanidins in modulation of carcinogen metabolism, providing new clues for better understanding the complex interaction between flavonoids and gut microbiota in cancer 145. PMFs prevented benzo[*a*]pyrene (B*a*P)/DSS-induced colorectal tumor formation in mice 146. PMFs increased the abundance of butyrate-producing probiotics (e.g., *Blautia* spp.) and decreased the levels of CRC-related bacteria (e.g., *Lactobacillus ruminis*). PMFs repressed the production of mutagenic metabolites of B*a*P and promoted B*a*P detoxification by modulating its colonic metabolism and xenobiotic-metabolizing enzyme (XME) expression. PMF was a promising chemopreventive agent that inhibited carcinogen bioconversion and improved gut microbiota homeostasis.

Anthocyanins are the glycosylated form of anthocyanidins. Anthocyanins are a subcategory of the flavonoid class that are water-soluble pigments responsible for the characteristic color of many fruits. Anthocyanins were capable of reducing tumor multiplicity in a mouse model of colon cancer 147. Anthocyanins could inhibit the proliferation, migration and colony formation of colon cancer cells *in vitro*. Mechanistically, anthocyanins enhanced the population of beneficial intestinal bacteria in the AOM/DSS murine model of colon cancer, which included *E. rectale*, *F. prausnitzii* and *Lactobacillus*. *E. rectale* and *F. prausnitzii* are butyrate-producing bacteria in the intestine 148. Butyrate is a SCFA produced by colonic fermentation of unabsorbed carbohydrate. Butyrate favors epithelial cell differentiation, abrogates inflammation and accelerates colon tissue repair. Probiotic *Lactobacillus* was reported to prevent colorectal carcinogenesis 149. By contrast, anthocyanins inhibited the growth of intestinal pathogenic microbiota including *Bacteroides*, *Campylobacter*, *H. pylori* and *Prevotella*. Altogether, anthocyanins markedly ameliorated gut microbiota dysbiosis, which induced aberrant epigenetic changes and deteriorated inflammation in AOM/DSS-treated mice. Likewise, the daily supplement of bilberry anthocyanin extracts inhibited the growth of colon adenocarcinomas and enhanced the therapeutic efficacy of anti-programmed cell death protein-1 (anti-PD-1) *in vivo* 10. Bilberry anthocyanin extracts elevated the abundance of the obligate anaerobe Clostridia and *Lactobacillus johnsonii*. Clostridia-produced butyrate might provide the energy for the survival and growth of other intestinal bacteria. Due to their strong antioxidant activity, the intake of dietary anthocyanins could enhance the consumption of intestinal oxygen. Thus, anthocyanins were assumed to manipulate intestinal microbiota by supplying energy for certain bacterial growth and/or by modulating the intestinal anaerobic environment through oxygen exhaustion. Consistently, the depletion of gut microbiota by an antibiotic cocktail abolished the synergic effect of bilberry anthocyanin extracts in combination with anti-PD-1 treatment. Thus, the establishment of a favorable intestinal microbiota by anthocyanins is conducive to the improvement in therapeutic efficacy of immune checkpoint inhibitors. Anthocyanin-rich extracts (AREs) from bilberry, chokeberry and grape reduced the number of large aberrant crypt foci (ACF) and inhibited colon carcinogenesis in rats treated with AOM 150. Mechanistic investigation indicated that AREs downregulated the levels of colonic mucosal cyclooxygenase-2 (COX-2) and fecal bile acids in rats by regulating intestinal microbial metabolism. Collectively, these findings supported the chemopreventive potential of anthocyanin.

Compared to gut microbiota in normal mice, there was a reduction of Bacteroidetes and an expansion of Firmicutes during the development of colitis-associated CRC (CAC) 151. ISL, a natural chalcone derived from licorice, was shown to reverse this imbalance at the phylum level. Thus, ISL prevented disease-induced alterations in the configuration of gut microbiota. Specifically, ISL caused a decrease in the abundance of *Helicobacteraceae*, and promoted the growth of *Lachnospiraceae* and *Rikenellaceae*. Increased abundance of *Lachnospiraceae* and *Rikenellaceae* might regulate the gut environment and reinforce the anticancer effects of ISL. Further, ISL exposure led to reduced levels of opportunistic pathogens (*Escherichia* and *Enterococcus*) and elevated amounts of butyrate-producing bacteria (*Butyricicoccus*, *Clostridium* and *Ruminococcus*). *Butyricicoccus* improved intestinal epithelial barrier function and protected the GIT of CAC patients. It was likely that *Clostridium* and *Ruminococcus* were involved in maintaining intestinal microbial balance. As a result, ISL protected mice from AOM/DSS-induced CAC. Altogether, ISL exerted anticancer effects in CAC by regulating the intestinal microbiota. EGCG was capable of reducing tumor load in a mouse model of AOM/DSS-induced CRC 152. EGCG increased the population of probiotics, such as *Bifidobacterium* and *Lactobacillus*. Probiotics may exert antagonistic effects on cancer. *Bifidobacterium* and *Lactobacillus* inhibited intestinal inflammation and carcinogenesis 153. Accordingly, the enrichment of probiotics was proposed as a mechanism for EGCG's chemopreventive effects.

Neohesperidin, a flavanone glycoside derived from citrus fruits, prevented colorectal tumorigenesis in the *Apc*^Min/+^ transgenic mouse model 154. The human gut microbiota is mainly dominated by Bacteroidetes (20%-40%) and Firmicutes (30%-50%) 155. Enrichment of Bacteroidetes and downregulation of Firmicutes and Proteobacteria have been closely connected with colorectal carcinogenesis 156. Neohesperidin triggered the enrichment of Firmicutes and Proteobacteria while decreased the relative abundance of Bacteroidetes 154. Moreover, neohesperidin-mediated apoptosis induction and angiogenesis inhibition could be abolished by antibiotic treatment. Thus, alternations of gut microbiota were required for the anticarcinogenic activity of neohesperidin.

*F. nucleatum* has been proven to play a role in CRC onset and progression. The previous study indicated that *F. nucleatum* promoted chemoresistance in CRC patients by activating the autophagy pathway 157. Dihydromyricetin (DHM), a natural flavonol, remarkably modified the composition and diversity of gut microbiota 158. DHM enhanced the chemotherapeutic efficacy of irinotecan by lowering the abundance of gut *Fusobacterium* in the mouse model of colitis-associated colon cancer. Another study demonstrated that DHM enriched the population of *Bacteroides thetaiotaomicron*, *Bifidobacterium*, *F. prausnitzii* and *Lactobacillus* 159. DHM elevated the expression of gut chloride channel 3 (CLCN3), which correlated with gut microbiota. In addition, DHM supplementation decreased the levels of butyrate at the hyperproliferative stage before tumor formation in AOM/DSS mice. Consequently, DHM modified the gut microbiota structure and decreased susceptibility to CRC carcinogenesis. Probiotic *Lactobacillus casei* prevented intestinal carcinogenesis by altering CLCN3 expression 160. DHM manipulated the tumor microenvironment that tended to recruit probiotics through upregulation of CLCN3. A previous study showed that loss of cystic fibrosis transmembrane conductance regulator (CFTR) resulted in the imbalance in gut ecosystem 161. DHM also relieved AOM/DSS-induced gut microbiota dysbiosis by upregulating CFTR. As a result, DHM could reduce susceptibility to colonic carcinogenesis caused by AOM/DSS in mice 159.

*Parabacteroides* inhibited AOM-driven colon tumor formation by blocking the Akt and TLR4 signaling cascades 162. Quercetin restrained tumor growth and reduced the mortality rate in CRC mice by increasing the relative levels of *Parabacteroides* 163. Quercetin was reported to reduce the levels of fecal bile acids in rats 164. It also overtly elevated the levels of betaine, fumarate and hippurate, while lowered the levels of creatinine. Therefore, quercetin exerted regulatory effects on several metabolic pathways including bile acid and amino acid metabolism. Thus, quercetin exhibited prominent bioactivity and play a critical role in altering gut microbiota, which might contribute to their anticancerous potential. Quercetin metabolites have been explored for their chemopreventive effects against cancers. The microbiota-derived metabolite of quercetin, 3,4-dihydroxyphenylacetic acid, repressed hemin-induced malignant transformation in colon cancer and colon epithelia cells 165. Mechanistically, 3,4-dihydroxyphenylacetic acid counteracted the promotive effects of hemin on cell proliferation, ROS production, DNA oxidative damage and mitochondrial dysfunction in colon cancer and colon epithelia cells. Likewise, the metabolites of quercetin from *Clostridium perfringens* and *B. fragilis* prevented the proliferation of colon cancer cells 166. The fermentation of human intestinal bacteria could enhance the tumor-suppressive effects of quercetin on cancer cells. It was inferred that microbial metabolites of quercetin were the major contributor to the chemopreventive benefit of quercetin *in vivo*. However, the cytotoxicity of purified metabolites of quercetin toward colon cancer cells remained equivocal. Additional work is required to define the protective role of the separate metabolites in cancer. Baicalin, the main constituent in the root of *Scutellaria baicalensis*, was rapidly converted into baicalein by intestinal microbiota 167. Baicalein significantly prohibited CRC development in a *Apc*^Min/+^ mice model by suppressing gut inflammation and inducing tumor cell death. Intriguingly, baicalein showed stronger anti-CRC activities than its parent compound baicalin. Gut microbiota might have the potential to magnify the tumor-inhibiting action of flavonoids, stressing the importance of the interplay between flavonoids and gut microbiota in controlling carcinogenesis. In addition, it remains to determine whether baicalin or baicalein can affect the intestinal microbiota structure.

Apigenin significantly restrained cancer cell growth and metastasis in the murine CRC model 168. Further investigation revealed that apigenin affected the composition of gut microbiota in mice. Specifically, apigenin treatment induced a decrease in the abundance of Firmicutes and an increase in the count of Actinobacteria. Firmicutes were found to generate oncogenic nitrogenous compounds via the decomposition of amino acids 155. The abundance of gut Actinobacteria might be inversely associated with CRC risk 156. Therefore, apigenin could ameliorate gut microbiota dysbiosis. The transplant of feces from mice treated with apigenin suppressed colon carcinogenesis in recipient mice, suggesting that apigenin-modulated gut microbiota exerted antitumor effects 168. These findings reinforced the relationship between the anticarcinogenic effect of apigenin and modulation of gut microbiota. Nevertheless, the exact mechanisms by which apigenin reshaped gut microbiota were not well understood. Continual efforts should be made to further understand the mechanistic association of apigenin, gut microbiota and colon cancer prevention.

The isoflavone curcumin, a bioactive component derived from the rhizome of *Curcuma longa*, is a lipophilic polyphenol that has been widely used as dietary spice 169. In recent years, curcumin has attracted great attention for its pharmacological activities. CRC progression was prone to correlate with the growth of *Prevotella* and *Ruminococcus* 153. The profiling of human gut microbiota demonstrated that curcumin could reduce the microbial abundance of these cancer-related species, suggesting its cancer chemopreventive role 170. Curcumin inhibited tumor growth in a mouse model of CAC 171. Curcumin markedly decreased the abundance of Clostridiales while increasing the levels of Bifidobacteriales, Coriobacteriales, Erisipelotrichales and Lactobacillales. It was postulated that the chemopreventive potential of curcumin was partially attributable to the expansion of Bifidobacteriales and Lactobacillales, which could prevent colorectal carcinogenesis. Curcumin prevented the occurrence of AOM-induced CRC in high-protein diet (HPD)-fed mice 172. In terms of mechanism, curcumin decreased the levels of colonic inflammatory proteins [e.g., COX-2 and iNOS (inducible nitric oxide synthase)] and fecal short- and branched-chain fatty acids. To summarize, curcumin impeded CRC development in an AOM-induced mouse model of colon carcinogenesis by limiting colonic inflammation and toxic metabolite secretion.

The secondary bile acids including deoxycholic acid and lithocholic acid show cytotoxic effects on normal colonic crypt cells, leading to colon carcinogenesis 173. The effects of dietary polyphenols on fecal secondary bile acids in rats fed a high-fat diet were previously explored 174. Curcumin remarkably diminished the fecal concentration of deoxycholic acid. Catechin and rutin significantly decreased the fecal concentration of lithocholic acid. Catechin, curcumin and rutin also reduced the fecal concentration of hyodeoxycholic acid, a metabolite of lithocholic acid. It was thus assumed that the chemopreventive effects of these polyphenols on the development of carcinogen-induced colon cancer was attributable to the downregulation of bile acids. The fruit of the date palm (*Phoenix dactylifera* L.) contains significant quantities of flavonoid glycosides (apigenin, kaempferol, luteolin and quercetin) and phenolic acids 175. The whole date fruit extract increased the growth of beneficial bacteria including *Bifidobacterium* and *Bacteroides*, and it also elevated the concentration of SCFAs (e.g., acetate, butyrate, lactate and propionate) following incubation with the fecal microbiota *in vitro* 176. The whole date fruit extract and its bacterial metabolite SCFAs exerted inhibitory effects on CRC cell growth. Continual investigations are warranted to confirm the anticancer mechanisms of action of flavonoid glycosides *in vivo*. Curcumin and the bisdemethoxycurcumin analog (BDMC-A) could efficiently block the incidence of 1,2-dimethylhydrazine (DMH)-driven colon carcinogenesis in rats 177. Mechanistically, the levels of fecal bile acids and cholesterol were declined in DMH-administered rats compared with control rats. The alterations in the fecal contents of bile acids and cholesterol in DMH-treated rats were significantly reversed by curcumin or BDMC-A administration. The concentration of colonic and intestinal cholesterol was markedly elevated, while that of phospholipid was decreased in DMH-driven tumor-bearing rats. Curcumin and BDMC-A overtly decreased the level of colonic and intestinal cholesterol and raised tissue phospholipid content. Curcumin and BDMC-A prevented DMH-induced colon carcinogenesis by regulation of cholesterol and phospholipid metabolism. *Astragalus mongholicus* Bunge-*Curcuma aromatica* Salisb. (ACE) was rich in four flavonoids (calycosin, calycosin-7-glucoside, formononetin, ononin) and three curcumins (bisdemethoxycurcumin, curcumin, demethoxycurcumin) 178. ACE restricted the overgrowth of pathogenic intestinal microbes, including *Escherichia-Shigella*, *Enterococcus* and *Streptococcus*, while the abundance of probiotic intestinal bacteria such as *Lactobacillus*, *Mucispirillum*, *Prevotellaceae*_*UCG-001* and *Roseburia* was increased. ACE elevated the fecal contents of butyric acid and propionic acid, resulting in restoration of the intestinal barrier integrity and suppression of CRC progression. Thus, ACE exhibited anticarcinogenic activities against CRC by modifying gut microbiota and regulating SCFA level.

The imbalance in gut microbiota is deemed as a crucial risk factor for CRC. Increasing experimental and preclinical evidence suggests that dietary flavonoids can prevent CRC development and progression, owing to their capability to restore gut microbiota homeostasis. In the future, more studies should be conducted to unravel the mechanisms by which pathologic changes in gut microbiota induce CRC formation, thus posing a potential target for cancer chemoprevention. Particularly, the roles of gut microbiota in the complicated signaling cascades associated with intestinal inflammation and carcinogenesis warrant further investigation. Gut microbiota can transform flavonoids into bioactive metabolites that can be easily absorbed by the human body. Therefore, more efforts are needed to adequately examine the protective effects of flavonoid metabolites against CRC. It is considered that gut microbiota composition and function may impact the biosynthesis of flavonoid metabolites. Due to inter-individual heterogeneity in responses to flavonoid consumption, it will be of great importance to identify the key factors that affect the flavonoid-gut microbiota interaction *in vivo*. Integrative analyses of microbial transcriptome, metagenomics and metabolomics will be essential in determining specific intestinal microbes that are responsible for the generation of bioactive flavonoid metabolites or are affected by flavonoid consumption. In-depth investigation on flavonoid metabolism will provide valuable clues on how flavonoid-induced alterations in gut microbiota contribute to CRC prevention.

## Flavonoids, gut microbiota and gastric cancer

The flavonoid compounds baicalin and baicalein showed an inhibitory effect against *H. pylori* and cytotoxicity toward gastric cancer cells 179. Baicalin and baicalein could repress the expression of *H. pylori* virulence factor VacA and prevented the adhesive and invasive abilities of *H. pylori* to gastric cancer cells. As baicalein, the aglycone form of baicalin, had the ability to penetrate into cells, it was possible that baicalein blocked *H. pylori* infection by directly acting on gastric cancer cells. This hypothesis remains to be validated in further studies. Notably, baicalin and baicalein suppressed the growth of *H. pylori* in the mice infection model. Accordingly, the serum levels of *H. pylori-*specific IgM and IgA were diminished in mice treated with baicalin and baicalein. Besides, baicalein exhibited a synergistic effect on abolishing *H. pylori* infections with *Lactobacillus* strains. The combination of baicalein and *Lactobacillus* strains had similar therapeutic effects as antibiotics but without disrupting the balance of gut microbiota. The efficacy and safety of baicalein/*Lactobacillus*-based *H. pylori* eradication therapy need to be validated in clinical trials. The flavonoid silibinin showed anti-*H. pylori* activities 180. Mechanistically, silibinin had the potential to interact with* H. pylori* Penicillin Binding Protein (PBP), causing its suppression and, thus, promoted significant morphological changes in the bacterial cell wall. Silibinin also interfered with *H. pylori*-stimulated immune responses*.* Consequently, silibinin showed antitumor activity against gastric adenocarcinoma cells. It could be concluded that silibinin might be effective in treating *H. pylori* infection and gastric cancer. Yeon et al. 181 found that kaempferol inhibited the translocation of CagA and VacA of *H. pylori* to gastric cancer cells by repressing the expression of bacterial secretion system components. At a result, kaempferol repressed the production of proinflammatory cytokines induced by *H. pylori* infection in gastric cancer cells. Collectively, kaempferol might prevent the development of gastric cancer by disturbing *H. pylori* infection-induced inflammatory response. The inhibitory effects of flavonoids on gastric cancer progression can be partially attributed to their antimicrobial actions against *H. pylori* 65. A growing body of evidence revealed that flavonoids could target various molecular targets in *H. pylori*, including cell membrane and wall, enzymes and secretion systems. However, most past studies only explored the anti-*H. pylori* property of flavonoids *in vitro* models. Despite the encouraging results achieved in previous studies, the mutual interactions between flavonoids and *H. pylori in vivo* during gastric cancer progression are worthy of further investigation.

## Flavonoids, gut microbiota and liver cancer

Polyvinylpyrrolidone-based solid dispersion of Zn(II)-curcumin (ZnCM-SD) repressed tumor growth and enhanced the tumor-suppressing effects of doxorubicin in a rat model of HCC by elevating the abundance of Bacteroidetes and decreasing the abundance of Firmicutes and the ratio of Firmicutes to Bacteroidetes 182. Depletion of gut microbiota abolished the anticancer effects of ZnCM-SD in combination with doxorubicin *in vivo*. Given the discrepancy in physiology and molecular targets between rats and humans, large prospective controlled clinical studies are indispensable to substantiate these encouraging results. O-desmethylangolensin (O-DMA) can be formed from daidzein by intestinal microbiota 183. O-DMA exhibited anticarcinogenic activity against HCC cells by inducing cell cycle arrest and promoting mitochondrial-dependent apoptosis 184. Xanthohumol, a prenylated flavonoid from hops, could be transformed into dihydroxanthohumol by the intestinal bacterium *Eubacterium ramulus* 185. Xanthohumol and dihydroxanthohumol exhibited antiproliferative abilities against liver carcinoma cells by inducing cell apoptosis through activation of caspases and promotion of cell membrane permeabilization 186. These plant-derived compounds showed promise as potential chemotherapeutic agents for the prevention and treatment of liver cancer.

The gut-liver axis gains much attention in recent years. The close anatomical, functional, bidirectional relationships between the GIT and liver, primarily through a portal circulation, contribute to the formation of the gut-liver axis. Gut microbiota is thought to have a strong relationship with the development, progression and complication of liver cancer. Therefore, it is necessary to develop alternative treatment options in attempt to control pathogenic factors involved in liver carcinogenesis within gut microbiota. Dietary modification is the focus for numerous studies aiming at manipulation of gut microbiota. Natural occurring flavonoids present chemopreventive and chemotherapeutic potentials by remodeling gut microbiota. However, there is limited literature on the efficacy and mechanisms of flavonoids in mitigating hepato carcinogenesis-associated intestinal dysbiosis. Experimental and clinical studies are required to confirm the impact of flavonoids on the gut-liver axis and the therapeutic potential of flavonoids in liver cancer.

## Flavonoids, gut microbiota and breast cancer

Genistein intake after tamoxifen therapy reduced the risk of local mammary cancer recurrence in rats fed with a high fat diet 187. Mechanistically, genistein lowered the abundance of inflammatory *Enterobacteriaceae* and *Prevotellaceae* while enriched the population of anti-inflammatory, SCFA-producing *Clostridiaceae*. Thus, genistein could reduce the risk of cancer recurrence by limiting inflammation. Genistein supplementation regulated gut metabolites, especially those associated with polyamine metabolism and pre-resolving phase of inflammation. Specifically, genistein elevated the levels of spermidine and phloretin, all of which were associated with reduced carcinogenesis 188, 189. Genistein reduced the levels of pro-tumorigenic metabolites, including N-acetylvaline, tyramine and trigonelline. The regulation of gut microbial metabolites might be a possible explanation for the inhibition of tumor recurrence by genistein. Another study reported that members of *Akkermansia*, *Eubacterium* and *Lactococcus* genera were significantly increased in humanized mice after consumption of the genistein diet, while there was a decrease in the amounts of *Anaerostipes*, *Bacteroides*, *Blautia*, *Coprobacillus*, *Paraprevotella* and *Turicibacter* genera 190. The growth of breast cancer cells was markedly inhibited in the humanized mice fed with genistein prior to tumor induction. Collectively, the modulation of intestinal microbiota may underlie the mechanisms of action of genistein. The influence of gut microbiota may extend beyond the gut through induction of metabolic changes. Gut microbiota orchestrates estrogen formation via secretion of β-glucuronidase, an enzyme that can converse estrogens into their active forms 191. The dysbiosis of gut microbiota damages the biotransformation of estrogen by intestinal microbes, leading to a decrease in circulating estrogens. The deregulation of circulating estrogens may drive breast carcinogenesis. Further research efforts are warranted to elucidate the direct relationship between gut microbiota dysbiosis and breast carcinogenesis. Moreover, genistein consumption enriched the population of SCFA-producing bacteria, including *Lachnospiraceae* and *Ruminococcaceae* 190. Genistein and SCFAs were found to prevent carcinogenesis via an epigenetic mechanism 192. In genistein-fed humanized mice, increased production of genistein metabolites and SCFAs might induce epigenetic changes contributing to the effectiveness of genistein in breast tumor inhibition. A thorough exploration is necessary to figure out whether the antagonistic action of genistein in breast cancer development is a consequence of its impact on the metabolic profile.

Microbial metabolites of flavonoids play an important role in breast cancer chemoprevention. The gut metabolites of anthocyanins and ellagitannins showed *in vitro* antiproliferative activity against breast cancer cells 193. The inhibition of breast cancer cell proliferation could be attributable to synergistic effects among the metabolites of anthocyanins and ellagitannins (e.g., phenolic acids and urolithins). Several mechanisms were implicated in the chemopreventive potential of these metabolites, which included blockade of aromatase activity, regulation of the positive-estrogen receptor, induction of apoptosis and activation of the H2AX and PI3K pathways. Daidzein and its metabolite equol strongly inhibited the activity of breast cancer resistance protein (BCRP) and, thus, enhanced the chemosensitivity of breast cancer cells towards BCRP substrates 194. S-equol suppressed the proliferation and promoted the apoptosis of breast cancer cells 195. Mechanistically, S-equol upregulated miR-10a-5p and inhibited the expression of its downstream target PI3K p110α. This event caused the inactivation of Akt protein and thereby enhanced the expression of apoptosis-related proteins. O-DMA promoted the apoptosis and inhibited the proliferation of breast cancer cells 196. O-DMA induced cell cycle arrest by regulating the interaction between the cyclin-dependent kinase (CDK) 4/6-cyclin D and CDK1-cyclin B complexes. The regulation of cell cycle regulators might constitute a major mechanism responsible for the anticancer property of O-DMA. In summary, flavonoid metabolites have shown potential to effectively act as anticancer agents *in vitro*, emphasizing the need for *in vivo* assessment of their anticancer efficacy in further studies.

## Flavonoids, gut microbiota and urogenital system tumor

Gut microbiota converted flavanols into bioactive metabolites that were mainly excreted via urine 11. Uroepithelial cells are thus exposed to high concentrations of flavonoid metabolites. Particularly, microbial metabolites (hippuric acids, phenylalkyl acids and valerolactones) of flavanols showed antiproliferative activities against bladder cancer cells. Therefore, microbial metabolites of flavanols might be responsible for chemoprevention in uroepithelial cells. There were pronounced individual differences in urine profiles arising from flavanol consumption. As expected, the urine compounds exhibited distinct antiproliferative activities against bladder cancer cells. Individual genetics and gut microbiota profiles might have an impact on flavonoid metabolism, thus affecting the composition and activity of flavonoid metabolites. These findings strengthened the notion that a better comprehension of flavonoid metabolism profiles facilitated the design of personalized adjuvant therapy regimens for cancer prevention. Further research is required to identify specific flavanol metabolites that exert anticancerous effects. Gut microbiota-derived metabolites of ellagitannins and green tea catechins, urolithin A (uroA) and 5-(3', 4', 5'-trihydroxyphenyl)-γ-valerolactone (M4), showed synergistic antiproliferative effects on prostate cancer cells 197. The dysregulation of androgen receptor (AR) and PI3K/Akt signaling cascades is related to the development of prostate cancer. It was worth noting that M4 further prevented the secretion of prostate-specific antigen (PSA) and promoted AR cytoplasmic retention caused by uroA. M4 showed the potential to block the PI3K/Akt pathway via inhibition of Akt phosphorylation. However, uroA remarkably promoted Akt phosphorylation, which might be due to the significant suppression of AR activity in uroA-treated prostate cancer cells. These results demonstrated that colonic metabolites of ellagitannins and catechins were propitious to chemoprevention of prostate cancer. Unlike uroA, M4 seemed to have limited contribution to the deregulation of AR and PI3K/Akt signaling pathways. Thus, the mechanisms responsible for M4's cytotoxic activity against prostate cancer cells have not been fully disclosed and require continued study. Several catechin metabolites (EGC-M2, EGC-M7 and EGC-M9) produced from epigallocatechin and EGCG by intestinal microbiota exhibited antiproliferative effects on cervical cancer cells 198. These metabolites might be responsible for the anticancer effect of green tea or EGCG. It was inferred that three adjacent hydroxyl groups of the phenyl moiety in the chemical structures of these metabolites were critical for their antiproliferative activities. Epicatechin metabolite EC-M9, which harbored only two adjacent hydroxyl groups in the phenyl moiety, also inhibited the proliferation of cervical cancer cells. This observation suggested that aliphatic side chain, valeric acid, played a key role in conjunction with two adjacent hydroxyl groups in the phenyl moiety. Expectedly, EGC-M9 encompassing both three adjacent hydroxyl groups in the phenyl moiety and valeric acid displayed the strongest anticancer property. The *in vivo* effects of these metabolites are worthy of further verification. The chemical structures of flavonoid metabolites may impact their affinity for cancer cells, accounting for distinct anticancerous capabilities of these metabolites. The anticancer mechanisms of flavonoid metabolites remain to be fully deciphered.

## Flavonoids, gut microbiota and other cancers

Icariside I, a prenylated flavonoid isolated from *Epimedium*, apparently suppressed tumor growth in a melanoma-bearing mouse model 199. Mechanistically, oral administration of icariside I increased the abundance of *Bifidobacterium* spp. and *Lactobacillus* spp. in the ceca of melanoma-bearing mice. Icariside I promoted the generation of gut microbiota-derived metabolites including indole derivatives and SCFAs, hence facilitating the restoration of intestinal barrier function and alleviating system inflammation in mice. In addition, icariside I showed immunological antitumor capability by strikingly increasing the population of various lymphocyte subsets in peripheral blood of tumor-bearing mice. Clinical studies should be conducted to evaluate the anticancer efficacy of Icariside I. Anthocyanin and its microbial metabolite protocatechuic acid (PCA) were effective in suppressing *N*-nitrosomethylbenzylamine (NMBA)-induced esophageal tumorigenesis in rats by inhibiting the expression of inflammation markers, including soluble epoxide hydrolase (sEH), COX-2 and iNOS 200. PCA can be easily synthesized and is more stable than anthocyanin. PCA may represent a promising chemopreventive agent in the treatment of esophageal cancer. Urolithin A and B are prevalent metabolites produced from the transformation of ellagitannins through intestinal microbes 201. Urolithin A and B could modify leukemic cell metabolism, as evidenced by elevated metabolic rate and significant alterations in glutamine metabolism, lipid metabolism and one-carbon metabolism. These events resulted in the inhibition of proliferation and the induction of apoptosis in leukemic cells. Collectively, urolithin A and B exhibited an inhibitory effect on leukemic cell proliferation by inducing shifts in cellular energy metabolism beneficial for adaptation to oxidative stress and promotion of apoptosis. The anti-leukemic action of urolithins *in vivo* deserves further study.

## Conclusions and future perspectives

In this review, we highlighted the key roles of flavonoids in modulation of intestinal microbiota with the purpose of providing new insights into the molecular mechanisms of action of flavonoids in cancer. Study of gut microbiota has become the new frontier, and our knowledge of the composition and functions of human gut microbiota has exponentially expanded in the past few years. Nevertheless, mechanistic investigations aiming to elucidate how gut microbiota affect cancer progression are still at the early stage, mainly revealing a linkage rather than a causal relationship. Efforts are still needed to profoundly define the causative role of gut microbiota in cancer development. Gut microbiota could become a significant component of cancer prevention and treatment in the future. Although some intestinal microbes elevate the risk of cancers, certain beneficial microbial species can protect against various cancers, potentially by converting dietary components into bioactive metabolites 16. The beneficial gut microbiota has a synergistic effect with chemopreventive and chemotherapeutic agents. The harmful microbiota can be diminished or eliminated to maintain the homeostasis of gut microbiota. Based on previous studies, the level and diversity of gut microbiota substantially differ between cancer patients and healthy controls. Therefore, gut microbiota may be used as therapeutic targets and predictive/prognostic biomarkers in cancer. Furthermore, gut microbiota serves as a critical mediator for the diet-cancer linkage. It is imperative to thoroughly understand the beneficial microbiota-mediated anticancer mechanisms of dietary bioactive compounds, and to validate the therapeutic potentials of targeting gut microbiota by dietary components in randomized controlled clinical studies. In-depth investigation on gut microbiome will accelerate the translation of gut microbiota researches in clinical practice.

The imbalance of gut microbiota is linked with the occurrence and development of cancer. Accumulating evidence confirms that the anticancer property of flavonoids is due to their modulation of gut microbiota (Figure 3). Particularly, flavonoids increase the abundance of beneficial intestinal microorganisms and reduce the abundance of pathogenic species. Flavonoids may hold promise as novel agents to treat intestinal dysbiosis and cancer. These bioactive compounds reshape gut microbiota and offer the advance of more effective drugs for cancer treatment. It has been widely believed that regular consumption of flavonoids has multifaceted health benefits. Metabolism of flavonoids mainly occurs in the intestine. Long retention time of flavonoids in the intestine can enhance propitious effects on gut microbiota that in turn reinforce the biological function of flavonoids by converting them into bioactive metabolites. However, severe challenges remain to be tackled. Firstly, poor bioavailability of flavonoids has been a concern that makes it hard to achieve optimal efficacy. This issue limits the utilization of flavonoids in nutraceutical and functional foods for therapeutic purposes. Gut microbiota is known to play a vital role in the absorption and metabolism of flavonoids 120. Phase II metabolism has an impact on the bioavailability of flavonoids in humans. Flavonoids commonly undergo sulfation, methylation and glucuronidation in the small intestine, liver and colon. The resultant metabolites can be detected in plasma following flavonoid intake. Several measures, including microemulsions, enzymatic methylation of flavonoids, microencapsulation and nano-delivery systems, are proposed to improve the bioavailability and absorption of flavonoids. Reportedly, fat ingestion increases the bioavailability and intestinal absorption of flavonoids via enhanced secretion of bile salts which promote micellar incorporation of flavonoids 202. Extensive studies are warranted to further understand the bioavailability of flavonoids as it is an important determinant of their biological functions. Secondly, as the concentration of flavonoids differ in plants, it is essential to determine the suitable dosage of flavonoids with the ultimate aim of achieving the optimal therapeutic efficacy. Gut microbiota has the ability to biotransform flavonoid compounds into different metabolites that have anticancer properties. It is worth exploring whether the biological activity of flavonoid metabolites is the main reason behind flavonoid-mediated cancer chemoprevention *in vivo*. That is to say, the microbial metabolism of flavonoids and the anticancer mechanisms of action of flavonoids deserve further study. Because of interpersonal variability in gut microbiota configurations, flavonoid metabolites produced by intestinal microbes may vary between individuals, posing the necessity to develop flavonoid-oriented personalized adjuvant therapies to prevent cancer. Thirdly, although many *in vitro* and animal studies have validated the anticancer properties of flavonoids, there is a lack of *in vivo* studies involving humans on this topic. Different animal models and clinical studies are needed to comprehensively define the reciprocal relationship between dietary intake of flavonoids and gut microbiota, hence providing a better comprehension of health benefits and potential therapeutic efficacy of flavonoids. Fourthly, consumption of flavonoids markedly affects the growth of gut microbiota, suggesting the prebiotic benefit of flavonoids. The roles of flavonoids as prebiotics in the gut may vary depending on the inhabiting probiotic strains. Consumption of probiotics modifies the function of intestinal microbes and restricts the growth of cancer-causing microbial organisms 203. Given their antitumor efficacy, probiotics can be used to prevent cancer development. Consequently, combination of probiotics and flavonoid formulations may represent an effective therapeutic strategy against cancer. Collectively, flavonoids have demonstrated huge potential as candidates for the development of novel cancer chemopreventive agents. Further studies on flavonoids in respect to their effective dosages, enhanced bioavailability and efficacy via specific techniques, long-term toxicities, pharmacokinetics and exact molecular actions in pre-clinical and clinical studies are warranted before their commercial applications in drug industry.

## Figures and Tables

**Table 1:** Mechanisms of action of flavonoids and their metabolites in cancer

Compound	Source	Effect	Mechanism	Reference
Anthocyanidin	Bilberry	Inhibit the proliferation of colon cancer cells	Relieve metabolic shifts caused by gut microbiota dysbiosis; enhance carcinogen detoxification	145
Polymethoxyflavone	Citrus fruit	Prevent colorectal carcinogenesis	Increase the abundance of butyrate-producing probiotics (e.g., *Blautia* spp.); reduce the numbers of carcinogenic bacteria (e.g., *Lactobacillus ruminis*); promote carcinogen detoxification	146
Anthocyanin	Black raspberry	Inhibit the proliferation, migration and colony formation of colon cancer cells; reduce tumor multiplicity *in vivo*	Increase the abundance of *Eubacterium rectale*, *Faecalibacterium prausnitzii* and *Lactobacillus*; decrease the levels of *Bacteroides*, *Campylobacter*, *Helicobacter pylori* and *Prevotella*	147
	Bilberry	Enhance the antitumor efficacy of immune checkpoint inhibitors against colon cancer	Increase the abundance of Clostridia and *Lactobacillus johnsonii*	10
	Bilberry, chokeberry and grape	Suppress colon carcinogenesis	Decrease the levels of colonic mucosal cyclooxygenase-2 and fecal bile acids	150
	Native Brazilian cherry	Inhibit the proliferation and induce cell cycle arrest in breast cancer cells	Produce bioactive metabolites (e.g., phenolic acids) by gut microbiota	193
	Black raspberry	Prevent the development of esophageal cancer	Reduce the expression of inflammation markers (sEH and COX-2)	200
Isoliquiritigenin	Licorice	Reduce the incidence of colitis-associated colorectal cancer	Increase the abundance of Bacteroidetes, *Butyricicoccus*, *Clostridium*, *Lachnospiraceae*, *Rikenellaceae* and *Ruminococcus*; reduce the abundance of *Enterococcus*, *Escherichia*, Firmicutes and *Helicobacteraceae*	151
Epigallocatechin gallate	Green tea	Suppress the growth of colorectal cancer	Increase the abundance of *Bifidobacterium* and *Lactobacillus*	152
Neohesperidin	Citrus fruit	Prevent colorectal tumorigenesis	Increase the levels of Firmicutes and Proteobacteria; decrease the abundance of Bacteroidetes	154
Dihydromyricetin	Vine tea	Enhance the antitumor efficacy of irinotecan against colon cancer	Decrease the abundance of gut *Fusobacterium*	158
		Reduce susceptibility to AOM/DSS-induced colonic carcinogenesis	Increase the numbers of *Bacteroides thetaiotaomicron*, *Bifidobacterium*, *Faecalibacterium prausnitzii* and *Lactobacillus*; decrease the level of butyrate	159
Quercetin	Synthetic	Inhibit the growth of colorectal cancer	Increase *Parabacteroides* abundance	163
		Prevent malignant transformation and mitochondrial dysfunction in colon cancer	Produce the bioactive metabolite, 3,4-dihydroxyphenylacetic acid, by gut microbiota	165
		Inhibit colon cancer cell proliferation	Produce bioactive metabolites by gut microbiota	166
Baicalin	Chinese skullcap	Repress the growth of colorectal cancer and block gut inflammation	Produce the bioactive metabolite, baicalein, by gut microbiota	167
		Exhibit cytotoxicity toward gastric cancer cells	Attenuate the virulence of *Helicobacter pylori*	179
Apigenin	Citrus fruit	Suppress the growth and metastasis of colon cancer cells	Decrease the abundance of Firmicutes and increase the level of Actinobacteria	168
Curcumin	Turmeric	Inhibit the growth of colon cancer	Decrease the abundance of Clostridiales and increase the abundance of Bifidobacteriales, Coriobacteriales, Erisipelotrichales and Lactobacillales	171
		Impede the occurrence of AOM-induced colorectal cancer	Lower the levels of colonic inflammatory proteins (COX-2 and iNOS) and fecal short- and branched-chain fatty acids	172
		Block colonic carcinogenesis	Reduce the fecal concentration of deoxycholic acid and hyodeoxycholic acid	174
		Prevent colonic carcinogenesis	Increase fecal concentration of bile acids and cholesterol; decrease the level of colonic and intestinal cholesterol and raise tissue phospholipid content	177
		Suppress the growth of hepatocellular carcinoma; enhance the tumor-suppressing effects of doxorubicin	Enrich the population of Bacteroidetes and decrease the abundance of Firmicutes	182
Rutin	Tartary buckwheat	Block colonic carcinogenesis	Reduce the fecal concentration of lithocholic acid and hyodeoxycholic acid	174
Flavonoid glycosides (apigenin, kaempferol, luteolin and quercetin)	Date palm	Exert antiproliferative effects on colorectal cancer cells	Increase the growth of beneficial bacteria including *Bifidobacterium* and *Bacteroides*; enhance the concentration of SCFAs	176
Bisdemethoxycurcumin analog	Synthetic	Prevent colonic carcinogenesis	Increase fecal concentration of bile acids and cholesterol; decrease the level of colonic and intestinal cholesterol and raise tissue phospholipid content	177
*Astragalus mongholicus* Bunge-*Curcuma aromatica* Salisb. (calycosin, calycosin-7-glucoside, formononetin, ononin, bisdemethoxycurcumin, curcumin, demethoxycurcumin)	*Astragalus*, wild turmeric	Inhibit the growth and metastasis of colorectal cancer	Inhibit the growth of *Escherichia*-*Shigella*, *Enterococcus* and *Streptococcus*; increase the abundance of *Lactobacillus*, *Mucispirillum*, *Prevotellaceae*_*UCG-001* and *Roseburia*; elevate the fecal contents of butyric acid and propionic acid	178
Baicalein	Chinese skullcap	Exert cytotoxic effects on gastric cancer cells	Attenuate the virulence of *Helicobacter pylori*	179
Silibinin	Milk thistle	Show anticancer activity against gastric adenocarcinoma cells	Inhibit *Helicobacter pylori* growth	180
Kaempferol	*Ginkgo biloba*	Exert cytotoxic effects on gastric adenocarcinoma cells	Mitigate *Helicobacter pylori*-induced inflammation	181
O-desmethylangolensin	Soybean	Inhibit the proliferation of hepatocellular carcinoma cells	Induce cell cycle arrest and promote apoptosis in hepatocellular carcinoma cells	184
		Repress the proliferation and promote the apoptosis of breast cancer cells	Induce cell cycle arrest in breast cancer cells	196
Xanthohumol/Dihydroxanthohumol	Hop	Exhibit antiproliferative activity against hepatocellular carcinoma cells	Induce the apoptosis of hepatocellular carcinoma cells	186
Genistein	Soybean	Reduce the risk of local mammary cancer recurrence	Lower the levels of *Enterobacteriaceae* and *Prevotellaceae*; increase the abundance of *Clostridiaceae*	187
		Inhibit the growth of breast cancer cells	Elevate the counts of *Akkermansia*, *Eubacterium*, *Lachnospiraceae*,* Lactococcus* and* Ruminococcaceae*; reduce the abundance of *Anaerostipes*, *Bacteroides*, *Blautia*, *Coprobacillus*, *Paraprevotella* and *Turicibacter*	190
Ellagitannin	Native Brazilian cherry	Inhibit the proliferation and induce cell cycle arrest in breast cancer cells	Produce bioactive metabolites (e.g., urolithins) by gut microbiota	193
	Pomegranate	Inhibit the proliferation of prostate cancer cells	Produce the bioactive metabolite, urolithin A, by gut microbiota	197
Daidzein/equol	Soybean	Increase the chemosensitivity of breast cancer cells	Suppress the activity of the drug transporter, breast cancer resistance protein	194
S-equol	Soybean	Suppress the proliferation and promote the apoptosis of breast cancer cells	Upregulate miR-10a-5p and block the PI3K/AKT signaling pathway	195
Flavanol	Grape	Inhibit the proliferation of bladder cancer cells	Produce bioactive metabolites (hippuric acids, phenylalkyl acids and valerolactones) by gut microbiota	11
Catechin	Green tea	Inhibit colonic carcinogenesis	Decrease the fecal concentration of lithocholic acid and hyodeoxycholic acid	174
		Inhibit the proliferation of prostate cancer cells	Produce the bioactive metabolite, 5-(3', 4', 5'-trihydroxyphenyl)-γ-valerolactone, by gut microbiota	197
Epigallocatechin/ Epigallocatechin gallate	Green tea	Inhibit the proliferation of cervical cancer cells	Produce bioactive metabolites (EGC-M2, EGC-M7 and EGC-M9) by gut microbiota	198
Epicatechin	Green tea	Inhibit the proliferation of cervical cancer cells	Produce the bioactive metabolite, EC-M9, by gut microbiota	198
Icariside I	*Epimedium*	Restrain melanoma growth	Elevate the abundance of *Bifidobacterium* spp. and *Lactobacillus* spp.; promote the generation of gut microbiota-derived metabolites (indole derivatives and SCFAs)	199
Protocatechuic acid	Black raspberry	Prevent esophageal carcinogenesis	Lower the expression of inflammation markers (sEH, COX-2 and iNOS)	200
Urolithin A and B	Pomegranate	Restrain the proliferation of leukemic cells	Regulate cellular energy metabolism; modify glutamine metabolism, one-carbon metabolism and lipid metabolism	201
